# Indecent work perception and burnout among psychiatric nurses: the mediating role of emotion regulation

**DOI:** 10.3389/fpubh.2025.1710294

**Published:** 2026-01-05

**Authors:** Shusi Tang, Lu Wang, Aifei Pan, Peipei Zheng, Chaohao Xu, Yingna Jin, Zhengzhou Zhang, Changchang Lin

**Affiliations:** 1 Department of General Psychiatry, Wenzhou Seventh People's Hospital, Wenzhou, China; 2 Department of Operating Room, Wenzhou People's Hospital, Wenzhou, China; 3 Department of Emergency, Zhejiang Hospital, Hangzhou, China; 4 Department of Psychosomatic Medicine, Wenzhou Seventh People's Hospital, Wenzhou, China

**Keywords:** psychiatric nursing, burnout, indecent work perception, emotion regulation, cognitive reappraisal

## Abstract

**Background:**

Psychiatric nursing is characterized by high levels of occupational burnout, with indecent work perceptions emerging as a significant contributing factor. However, the psychological mechanisms underlying this relationship remain poorly understood. This study examined the mediating role of emotion regulation in the relationship between indecent work perception and burnout among psychiatric nurses.

**Methods:**

A cross-sectional survey was conducted in June 2024 among 195 psychiatric nurses from a psychiatric hospital in Wenzhou, China. Data were collected using self-report questionnaires assessing demographic characteristics, indecent work perception, emotion regulation, and burnout. Data analysis included descriptive statistics, correlation analysis, multiple linear regression, and structural equation modeling with bootstrapping procedures to test mediation effects.

**Results:**

The sample comprised psychiatric nurses with a mean age of 30.12 years and 8.04 years of occupational experience. All three dimensions of indecent work perception were positively associated with burnout, with working position concerns showing the strongest association (β = 0.39, *p* < 0.001). The overall model explained 61% of the variance in burnout scores. Mediation analysis revealed that cognitive reappraisal significantly mediated the relationship between indecent work perception and burnout [*indirect effect* = 0.02, *95% CI* (0.01, 0.03), *p* < 0.001], accounting for approximately 17% of the total effect. In contrast, expressive suppression did not demonstrate significant mediation [*indirect effect* = 0.001, *95% CI* (−0.002, 0.003), *p* = 0.87].

**Conclusions:**

Indecent work perception is an important risk factor for burnout among psychiatric nurses, and diminished use of cognitive reappraisal partly meditates this association. These findings highlight the need for organizational interventions that enhance recognition and career development opportunities, alongside individual-level strategies that strengthen adaptive emotion regulation skills. Addressing both contextual and psychological factors may be critical for reducing burnout and promoting sustainable professional development in psychiatric nursing.

## 1 Introduction

Psychiatric nursing is widely recognized as a profession under considerable psychological strain (1, 2). Occupational burnout—characterized by emotional exhaustion, depersonalization, and diminished personal accomplishment—is particularly prevalent in mental health settings. Psychiatric nurses, in particular, often work the longest hours among healthcare professionals (3). For example, Tang et al. (4) reported that 73.69% of psychiatric nurses experienced moderate to severe emotional exhaustion and depersonalization, with nearly all reporting reduced personal accomplishment. Likewise, a systematic review of mental health nurses found that approximately 25% reported high emotional exhaustion, 15% high depersonalization, and 22% low personal accomplishment (5). The Job Demands–Resources model offers a useful theoretical framework, suggesting that excessive emotional and cognitive demands, when unmitigated by resources such as social support or recognition, accelerate the depletion of energy and foster burnout (6). In psychiatric care, additional stressors—including patient aggression, social stigma, and ethical dilemmas—further intensify these demands, while organizational constraints frequently limit resource availability (7). Burnout among psychiatric nurses is not only a serious health issue but also undermines work outcomes by shaping employees' perceptions of job demands and resources, thereby contributing to absenteeism. Because psychiatric nurses themselves serve as therapeutic instruments for patients (8), they must maintain continuous close contact with individuals experiencing psychosis, suicidal ideation, or erratic behavior. This constant exposure exacerbates both physical and psychological strain, making them especially vulnerable to burnout.

Decent work is a concept introduced by the International Labor Organization, emphasizing fairness, dignity, and opportunities for professional development (9). In contrast, indecent work perception refers to the subjective experience that one's occupation lacks these qualities, and such perceptions have been shown to negatively affect occupational health and psychological wellbeing (10). Previous studies indicate that indecent or unfair work environments increase stress and erode professional identity, thereby elevating the risk of burnout. In psychiatric nursing, indecent work perception is particularly salient due to the specialty's unique professional challenges (11). On one hand, psychiatric nurses face persistent stigma from the public, patients' families, and even other healthcare professionals (12), who often undervalue mental health care compared with somatic specialties. On the other hand, career development in psychiatric nursing is frequently constrained not only by structural factors, such as limited promotion opportunities or access to specialized training, but also by perceptual barriers within daily clinical practice (13). For example, research by Jansen et al. (13) found that undergraduate nursing students perceived staff working in mental health settings as less competent. Such views reinforce broader stereotypes that psychiatric nursing offers fewer opportunities for skill development than specialties such as surgery or intensive care.

Although the association between indecent work perception and burnout has been increasingly acknowledged, the underlying psychological mechanisms remain insufficiently explored. Viewing work as indecent not only elicits negative emotions such as frustration, alienation, and hopelessness but also undermines individuals' capacity for emotion regulation (14). According to COR theory, perceiving the work environment as lacking dignity, fairness, or development opportunities constitutes a substantial resource threat that depletes the psychological reserves required for effective regulation (15). Prior studies have demonstrated that workplace injustice and devaluation are linked to reduced use of cognitive reappraisal and greater reliance on suppression, both of which predict heightened burnout and psychological distress (16). Consequently, indecent work perception may impair psychiatric nurses' ability to regulate emotions, establishing a mediated pathway through which negative occupational perceptions exacerbate the risk of burnout.

Despite increasing recognition that indecent work perception is an important predictor of burnout, limited research has examined the psychological processes through which this perception influences psychiatric nurses' occupational wellbeing. In particular, it remains unclear whether emotion regulation mediates this relationship. To address this gap, the present study examined the mediating roles of cognitive reappraisal and expressive inhibition in the association between indecent work perception and burnout among psychiatric nurses. Based on the Job Demands–Resources model and Conservation of Resources theory, we proposed the following hypotheses: H1: The perception of indecent work is positively associated with burnout in psychiatric nurses; H2: Emotional regulation strategies play a mediating role in the relationship between the perception of indecent work and burnout.

## 2 Method

### 2.1 Participants

Participants were recruited in June 2024 from a psychiatric hospital in Wenzhou City, China. A purposive sampling method was used to recruit participants who met the inclusion criteria. The inclusion criteria were: (1) being a certified nurse currently employed at the institution; (2) having adequate cognitive ability and literacy in Chinese to comprehend and complete the questionnaire; and (3) providing written informed consent prior to participation. Exclusion criteria included: (1) being on long-term leave or having recently resigned; (2) a documented history of severe mental disorders that could impair cognitive functioning; (3) refusal to complete the questionnaire or voluntary withdrawal during the survey process; and (4) inconsistent or suspicious response patterns suggestive of inattentive or invalid answering, including selecting the same response option across all items or displaying mechanical answering patterns (e.g., repeating sequential choices). After excluding cases with regular responses, 195 participants were included, among these, 181 were female (92.82%) and 14 were male. The mean age of participants was 30.26 years (*SD* = 5.63) with an average of 8.17 years of occupational experience (*SD* = 5.95). Regarding indecent work perceptions, working position concerns showed the highest mean score (*M* = 7.92), followed by career recognition (*M* = 7.49) and career development (*M* = 7.01) (see Table 1 for more details).

**Table 1:** Descriptive statistics of demographic characteristics, indecent work perception, emotion regulation, and burnout among psychiatric nurses.

**Variables**	**Mean**	**SD**	**Min**	**Max**	**Skewness**	**Kurtosis**
**Demography**						
Age	30.26	5.63	20.00	50.00	1.15	1.26
Occupational years	8.17	5.95	1.00	33.00	1.44	2.35
**Indecent work perception**						
Working position	7.64	2.83	3.00	15.00	0.28	−0.38
Career development	6.89	2.09	3.00	12.00	−0.23	−0.45
Career recognition	7.56	2.71	3.00	15.00	0.18	−0.19
**Emotion regulation**						
Cognitive reappraisal	30.39	6.07	18.00	42.00	0.49	−0.79
Expressive inhibition	16.93	4.84	4.00	28.00	0.26	0.56
**Burnout**	47.90	14.47	15.00	80.00	−0.55	−0.46

### 2.2 Procedures

Data was collected by an electronic questionnaire platform. A QR code linking to the online survey was generated and distributed via posters throughout the hospital. Head nurses in each ward assisted in identifying nursing staff who met the inclusion criteria. Prior to the formal launch of the survey, designated nursing staff in each ward received standardized training to ensure accurate understanding of all questionnaire items and clear enrollment criteria. This preparatory step aims to accurately enroll in-hospital psychiatric nurses and improve the reliability of data collection. Eligible participants were invited to complete the questionnaire by scanning the QR code in a quiet environment at their convenience.

### 2.3 Measurements

#### 2.3.1 Demographic information

Demographic information was collected using a self-compiled questionnaire. Variables included gender (male or female), age (in years), and occupational years (in years). Gender was coded as a binary variable, while age and occupational years were recorded as continuous variables.

#### 2.3.2 Indecent work perception

Indecent work perception was assessed using the Decent Work Perception Scale (DWPS) designed by Mao (17), from which three dimensions were selected: working position, career development, and career recognition. Each dimension was measured with three items, and some items were reverse scored to ensure response consistency. Higher scores reflected stronger perceptions of indecent work. In this study, the scale demonstrated good internal reliability (Cronbach's α = 0.86).

#### 2.3.3 Emotion regulation

Emotion regulation was measured using the Emotion Regulation Questionnaire (ERQ), a 10-item self-report scale that assesses individuals' habitual use of two strategies: cognitive reappraisal (6 items) and expressive inhibition (4 items) (18). Items were rated on a 7-point Likert scale ranging from 1 (“strongly disagree”) to 7 (“strongly agree”). Higher scores on each subscale indicated greater use of the corresponding strategy. In the present study, both subscales demonstrated satisfactory internal consistency (Cronbach's α = 0.90 for cognitive reappraisal; Cronbach's α = 0.89 for expressive inhibition).

#### 2.3.4 Burnout

Burnout was assessed using the Chinese version of the Burnout Scale revised by Li Chaoping et al. (19). This scale is widely applied in occupational health research in China and demonstrates good cultural adaptability and psychometric properties. Participants responded to items on a 7-point Likert scale, ranging from 0 (never) to 6 (daily). Higher total scores indicated greater levels of burnout. In this study, the scale showed good internal reliability (Cronbach's α = 0.92).

### 2.4 Statistical analysis

All statistical analyses were conducted using SPSS 26.0 (IBM Corp., Armonk, NY, USA) and R 4.4.1. Descriptive statistics, including means, standard deviations, skewness, and kurtosis, were calculated to examine the distributional characteristics of the study variables. The normality of distributions was evaluated using skewness and kurtosis criteria, with |skewness| < 2 and |kurtosis| < 7 considered acceptable (20). Pearson correlation analysis was performed to assess bivariate associations among indecent work perception, emotion regulation strategies, and burnout. To examine the predictive effects of independent variables on burnout, multiple linear regression analysis was conducted while controlling demographic variables (age, gender, and occupational years). Multicollinearity was assessed using variance inflation factors (VIF), with values less than 10 indicating no serious concerns (21). Finally, mediation analyses were performed using structural equation modeling in R package “lavaan,” with cognitive reappraisal and expressive inhibition specified as mediators in separate models. Indirect, direct, and total effects were estimated, and their significance was tested using bootstrap confidence intervals (*n* = 2,000). A two-tailed *p*-*value* < 0.05 was considered statistically significant.

## 3 Result

### 3.1 Descriptive statistics

Table 2 presents the correlations among the main study variables. The three dimensions of indecent work perception showed moderate to strong intercorrelations (*p* < 0.001). Burnout was positively correlated with working position (*p* < 0.001), career development (*p* < 0.001), and career recognition (*p* < 0.001). Cognitive reappraisal was negatively associated with career development (*p* < 0.001), career recognition (*p* < 0.001), and burnout (*p* < 0.001). Expressive inhibition was positively correlated with working position (*p* < 0.001 and with cognitive reappraisal (*p* < 0.001) but showed no significant relationship with burnout (*p* > 0.05).

**Table 2:** Correlation matrix of indecent work perception, emotion regulation, and burnout.

**Variables**	**1**.	**2**.	**3**.	**4**.	**5**.	**6**.	**7**.	**8**.	**9**.
1. Gender	(–)								
2. Age	0.12	(–)							
3. Occupational years	0.13^*^	0.91^***^	(–)						
4. Working position	−0.05	0.01	−0.02	(–)					
5. Career development	−0.08	0.11	0.08	0.50^***^	(–)				
6. Career recognition	−0.11	0.15^*^	0.14^*^	0.23^*^	0.55^***^	(–)			
7. Cognitive reappraisal	−0.05	−0.04	−0.01	−0.12	−0.40^***^	−0.49^***^	(–)		
8. Expressive inhibition	−0.10	−0.08	−0.16^*^	0.22^***^	0.06	−0.25^***^	0.35^***^	(–)	
9. Burnout	−0.11	−0.06	−0.12	0.56^***^	0.59^***^	0.50^***^	−0.52^***^	0.10	(–)

^*^p < 0.05; ^***^p < 0.001.

### 3.2 Multiple linear regression

To examine the unique contributions of indecent work perception and emotion regulation strategies in predicting burnout among psychiatric nurses, a multiple linear regression analysis was conducted with burnout as the dependent variable (Table 3). The overall model was statistically significant (*F* = 45.38, *p* < 0.001) and explained 61% of the variance in burnout (Adjusted *R*^2^ = 0.61), indicating substantial predictive power. Among the predictors, indecent work perception variables showed significant positive effects. Specifically, working position (β = 0.39, *p* < 0.001), career development (β = 0.14, *p* = 0.03), and career recognition (β = 0.20, *p* < 0.001) were positively associated with burnout. Regarding emotion regulation strategies, cognitive reappraisal demonstrated a significant negative association (β = −0.40, *p* < 0.001), whereas expressive inhibition was positively associated with burnout (β = 0.21, *p* < 0.001). In contrast, the control variables age, gender, and occupational years were not significant predictors of burnout.

**Table 3:** Linear regression model of associations of indecent work perception, emotion regulation, and demographic variables with burnout.

**Model**	**Variables**	**β**	***se***	***t***	***p***	***VIF***
Independent variable	Working position	0.39	0.05	7.76	< 0.001	1.50
	Career development	0.14	0.06	2.26	0.03	2.11
	Career recognition	0.20	0.06	3.56	< 0.001	1.81
	Cognitive reappraisal	−0.40	0.05	−7.27	< 0.001	1.72
	Expressive inhibition	0.21	0.05	4.07	< 0.001	1.57
Control variables	Age	0.04	0.10	0.39	0.70	5.96
	Gender	−0.01	0.04	−0.27	0.79	1.08
	Occupational years	−0.16	0.10	−1.55	0.12	6.05
*F*	45.38					
Adjusted *R*^2^	0.61					

### 3.3 Mediate path analysis

**Figure 1:**
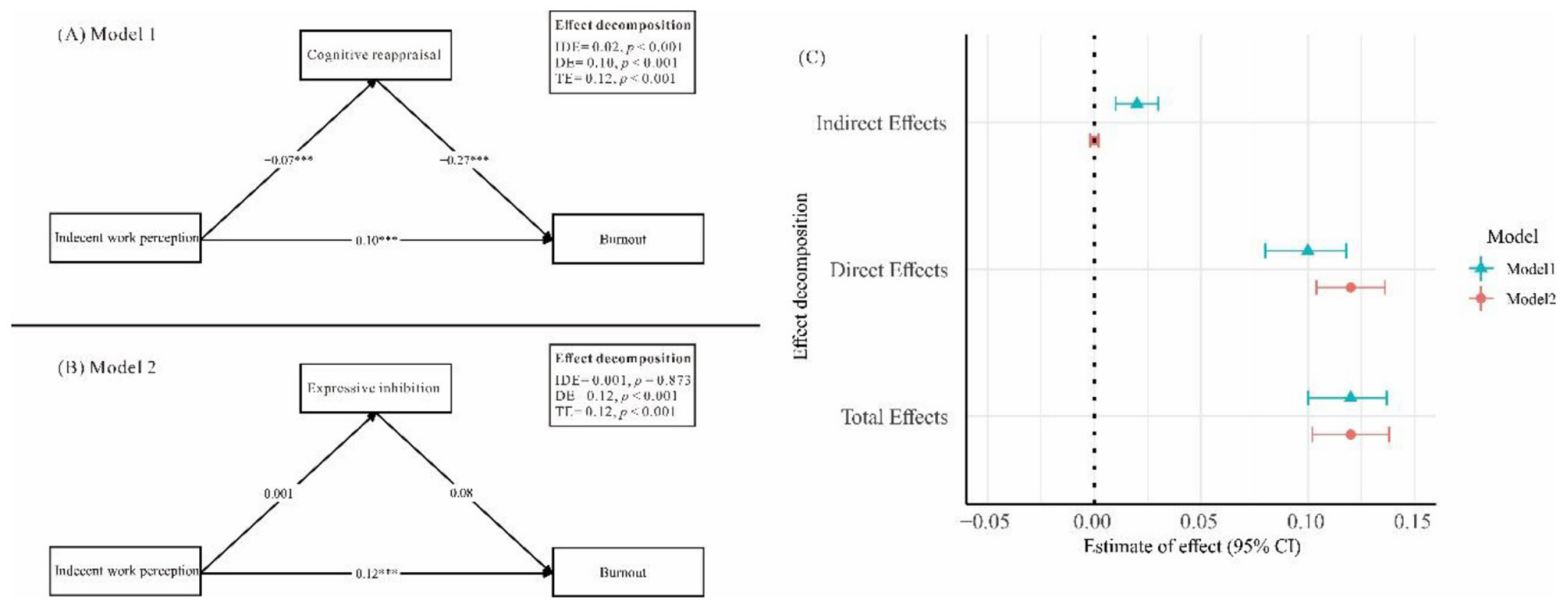
Mediation models of the relationship between indecent work perception and burnout through **(A)** cognitive reappraisal (Model 1) and **(B)** expressive inhibition (Model 2), with corresponding effect decomposition **(C)**.

The mediation effects of cognitive reappraisal and expressive inhibition on the relationship between indecent work perception and burnout were examined (Figure 1 and Table 4). For **Model 1**, cognitive reappraisal significantly mediated the association between indecent work perception and burnout. The indirect effect was significant (β = 0.02, *p* < 0.001), and both the direct effect (β = 0.10, *p* < 0.001) and total effect (β = 0.12, *p* < 0.001) remained significant. The indirect effect accounted for approximately 16.7% of the total effect, indicating a partial mediation in which cognitive reappraisal explained a meaningful proportion of the association between indecent work perception and burnout. In **Model 2**, expressive inhibition did not show a significant mediating effect. The indirect effect was non-significant (β = 0.001 *p* = 0.87). The direct effect (β = 0.12, *p* < 0.001) was nearly identical to the total effect (β = 0.12, *p* < 0.001), indicating that the effect of indecent work perception on burnout was almost entirely direct, with no meaningful contribution from expressive inhibition.

**Table 4:** Effect decomposition for the mediation model.

**Model**	**Effect**	***95%CI***	***z***	***p***
**Model 1**				
Indirect effect	0.02	[0.01, 0.03]	4.13	< 0.001
Direct effect	0.10	[0.08, 0.12]	11.33	< 0.001
Total effect	0.12	[0.10, 0.14]	14.07	< 0.001
**Model 2**				
Indirect effect	0.001	[−0.002, 0.003]	0.16	0.87
Direct effect	0.12	[0.11, 0.13]	14.15	< 0.001
Total effect	0.12	[0.10, 0.14]	14.07	< 0.001

## 4 Discussion

The present study aimed to investigate the mediating role of emotion regulation in the relationship between indecent work perception and burnout among psychiatric nurses. The main findings revealed that cognitive reappraisal significantly mediated the relationship between indecent work perception and burnout, accounting for approximately 17% of the total effect, while expressive suppression did not demonstrate significant mediation. Additionally, all three dimensions of indecent work perception (working position, career development, and career recognition) were significant predictors of burnout, with working position concerns showing the strongest association.

The result of multiple linear regression indicates that all three facets of indecent work perception—working position, career development, and career recognition—are independently and positively associated with burnout in psychiatric nurses, even after adjusting for demographics. This pattern reflects the unique occupational challenges inherent in psychiatric nursing, where nurses experience diminished professional recognition and less professional development compared to other nurses specialties (22). The prominence of working positions aligns with the COR theory, which suggests that when individuals perceive threats to their fundamental work resources—such as environmental safety, role clarity, and manageable workload—they experience resource depletion that leads to stress and eventual burnout (23). In psychiatric settings, these working position challenges are magnified by unpredictable patient behaviors and the emotional intensity of therapeutic relationships, creating a resource-depleting environment that is particularly conducive to burnout (24). The significant associations with career development and career recognition further underscore how psychiatric nursing's professional stigma and limited advancement opportunities compound the stress experienced by nurses. According to Social Identity Theory, when psychiatric nurses perceive their professional group as devalued or lacking in prestige (25), this threatens their professional identity and self-esteem, contributing to psychological distress and occupational dissatisfaction. In addition, the clinical particularity of psychiatric nursing amplifies these effects. Unlike many other medical specialties where treatment outcomes are more immediate and measurable, the recovery trajectory of patients with mental disorders is often slow, uncertain, and characterized by high relapse rates. The comparatively lower cure rate in psychiatric care may reduce the visibility of nurses' contributions and diminish their sense of professional accomplishment (13). This dynamic not only undermines occupational value but also reinforces perceptions of working in an “indecent” role. Thus, the combination of unfavorable structural conditions (working position, career development, career recognition) with clinical realities unique to psychiatric nursing creates a context in which indecent work perceptions strongly and consistently predict burnout.

Beyond the direct associations, this study further revealed that cognitive reappraisal mediated the relationship between indecent work perception and burnout, accounting for approximately 16.7% of the total effect. This suggests that perceiving work as indecent may weaken nurses' capacity to engage in adaptive cognitive reframing, thereby increasing vulnerability to burnout. From the perspective of COR theory, sustained experiences of undervaluation and limited development opportunities gradually erode psychological resources, leaving fewer cognitive and emotional reserves available for reappraisal. This is supported by Pálfi et al. (26), who found that emergency healthcare workers facing higher burnout reported less use of adaptive strategies like positive reappraisal. Similarly, repeated exposure to uncontrollable and demeaning work conditions reduces individuals' perceived efficacy in altering their emotional experiences, thus impairing their emotion regulation (27). Consistent with previous findings, studies have shown that healthcare workers exposed to low recognition and unfair treatment are less likely to employ reappraisal and more likely to rely on maladaptive coping mechanisms (28). Talebiazar et al. (29) found that nursing professionals who perceive a lack of recognition and control tend toward learned helplessness and reduced emotion regulation capacity, precipitating burnout. Extending these findings, Jin et al. (30) demonstrated among Chinese psychiatric nurses that cognitive reappraisal was negatively associated with burnout and buffered the effects of emotional labor, reinforcing the protective role of reappraisal. Taken together, these results indicate that indecent work perception undermines the use of cognitive reappraisal, creating an indirect pathway through which negative work evaluations accelerate burnout.

## 5 Conclusion

This study investigated the relationship between indecent work perception and burnout among psychiatric nurses, with a particular focus on the mediating role of emotion regulation strategies. The results demonstrated that indecent work perception was positively associated with burnout across its dimensions, and that cognitive reappraisal partially mediated this association, whereas expressive inhibition did not. These findings provide empirical support for theoretical frameworks such as the Job Demands–Resources model and conservation of resources theory, underscoring the importance of both contextual work perceptions and individual psychological resources in shaping burnout outcomes. From a practical perspective, the results suggest that interventions targeting both the work environment (e.g., enhancing professional recognition, career development, and support systems) and individual coping strategies (e.g., training in cognitive reappraisal) are effective in mitigating burnout among psychiatric nurses.

## 6 Implications

The findings of current study have several implications for clinical practice and organizational policy in psychiatric nursing. Given the strong association between indecent work perception and burnout, interventions should address both systemic and individual factors to promote occupational wellbeing. At the organizational level, healthcare institutions should focus on improving the structural conditions that contribute to perceptions of indecent work. This includes enhancing opportunities for career development, ensuring fair recognition of psychiatric nursing expertise, and fostering supportive leadership and teamwork climates. As highlighted by the Job Demands–Resources theory (6), increasing job resources such as recognition, autonomy, and professional growth opportunities can buffer the negative effects of high emotional demands and prevent resource depletion. Moreover, initiatives aimed at reducing professional stigma and reinforcing the value of psychiatric nursing can help strengthen nurses' professional identity and sense of accomplishment. At the individual level, interventions that enhance emotion regulation capacities may serve as effective tools to mitigate burnout. Training programs focusing on cognitive reappraisal, mindfulness, and stress management have demonstrated positive outcomes in reducing emotional exhaustion and improving coping among healthcare professionals (29). Integrating such courses into continuing professional development programs could equip psychiatric nurses with adaptive cognitive strategies to manage emotionally demanding situations.

## 7 Limitation

Several limitations should be acknowledged when interpreting the findings of this study. First, the cross-sectional design limits causal inference; longitudinal or experimental studies are needed to clarify the temporal ordering of indecent work perception, emotion regulation, and burnout. Second, all data were collected via self-report questionnaires, which may introduce social desirability or recall bias. Incorporating multi-method approaches, such as supervisor ratings or physiological indicators of stress, could strengthen the validity of future research. Third, the study sample was drawn from a single psychiatric hospital in Wenzhou, China, which may restrict the generalizability of results to other regions, healthcare institutions, or cultural contexts. Fourth, the sample was predominantly female, limit the generalizability of the findings to male nurses. Future research should seek to include a more gender-balanced sample where possible. Finally, the study examined only two emotion regulation strategies—cognitive reappraisal and expressive inhibition; other potentially important psychological mechanisms, such as resilience, mindfulness, or social support, were not included.

## Data Availability

The raw data supporting the conclusions of this article will be made available by the authors, without undue reservation.
